# An accurate test for homogeneity of odds ratios based on Cochran’s *Q*-statistic

**DOI:** 10.1186/s12874-015-0034-x

**Published:** 2015-06-10

**Authors:** Elena Kulinskaya, Michael B Dollinger

**Affiliations:** School of Computing Sciences, University of East Anglia, Norwich, NR4 7TJ UK; Department of Mathematics, Pacific Lutheran University, Tacoma, 98447 USA WA

**Keywords:** Meta-analysis, 2×2 tables, Heterogeneity test, Interaction test, Fixed effect model, Random effects model

## Abstract

**Background:**

A frequently used statistic for testing homogeneity in a meta-analysis of *K* independent studies is Cochran’s *Q*. For a standard test of homogeneity the *Q* statistic is referred to a chi-square distribution with *K*−1 degrees of freedom. For the situation in which the effects of the studies are logarithms of odds ratios, the chi-square distribution is much too conservative for moderate size studies, although it may be asymptotically correct as the individual studies become large.

**Methods:**

Using a mixture of theoretical results and simulations, we provide formulas to estimate the shape and scale parameters of a gamma distribution to fit the distribution of *Q*.

**Results:**

Simulation studies show that the gamma distribution is a good approximation to the distribution for *Q*.

**Conclusions:**

Use of the gamma distribution instead of the chi-square distribution for *Q* should eliminate inaccurate inferences in assessing homogeneity in a meta-analysis. (A computer program for implementing this test is provided.) This hypothesis test is competitive with the Breslow-Day test both in accuracy of level and in power.

**Electronic supplementary material:**

The online version of this article (doi:10.1186/s12874-015-0034-x) contains supplementary material, which is available to authorized users.

## Background

The combination of the results of several similar studies has many applications in statistical practice, notably in the meta-analysis of medical and social science studies and also in multi-center medical trials. An important first step in such a combination is to decide whether the several studies are sufficiently similar. This decision is often accomplished via a so-called test of homogeneity. The outcomes of the studies may be expressed in a variety of effect measures, such as: sample means; odds ratios, relative risks or risk differences arising from 2×2 tables; standardized mean differences of two arms of the studies; and many more. A variety of statistics for use in tests of homogeneity have been proposed; some are specific to the type of effect measure, and some are applicable to several measures.

This paper has its main focus on the test statistic first introduced by Cochran [1] and [2] and its application to testing homogeneity when the effects of interest are odds ratios arising from experiments with dichotomous outcomes in treatment and control arms. Cochran’s *Q* statistic is defined by $Q=\sum _{i} \widehat {w}_{i}(\widehat {\theta }_{i}-\widehat {\theta }_{w})^{2}$ where $\widehat {\theta }_{i}$ is the effect estimator of the *i*th study, $\widehat {\theta }_{w} =\sum _{i}\widehat {w}_{i}\widehat {\theta }_{i}/\sum _{i}\widehat {w}_{i}$ is the weighted average of the estimators of the effects, and the weight $\widehat {w}_{i}$ is the inverse of the variance estimator of *i*th effect estimator. The use of inverse variance weights has the appealing feature of weighting larger and more accurate studies more heavily in the weighted mean $\widehat {\theta }_{w}$ and in the statistic *Q*. This statistic was investigated for the case that the study effects are normally distributed sample means by Cochran and also by Welch [3] and James [4]. Perhaps the first application of the *Q* statistic to testing homogeneity of the logarithm of odds ratios is due to Woolf in 1955 [5]. DerSimonian and Laird [6] extended the use of *Q* for studies with binomial outcomes to difference of proportions as well as to log odds ratios in the context of the random effects model in which the studies are assumed to be sampled from a hypothetical population of potential studies. However, the use of *Q* in a test of homogeneity is the same whether a random effects or fixed effects model is used.

Under fairly general conditions, in the absence of heterogeneity, *Q* will follow asymptotically (as the individual studies become large) the chi-square distribution with *K*−1 degrees of freedom where *K* is the number of studies. It is common practice to assume that *Q* has this null distribution, regardless of the sizes of the individual studies or the effect measure. But this null distribution is inaccurate (except asymptotically), and its use causes inferences based on *Q* to be inaccurate. This conclusion of inaccuracy should also apply to inferences based on any statistics which are derived from *Q*, such as the *I*^2^ statistic (see [7] and [8]). Little is known of a theoretical nature about the null distribution of *Q* under non-asymptotic conditions. In our previous work, together with Bjørkestøl, we have provided improved approximations to the null distribution of *Q* when the effect measure of interest is the standardized mean difference [9] and the risk difference [10]. In this paper we use a combination of theoretical and simulation results to estimate the mean and variance of *Q* when the effects are logarithms of odds ratios. We use these estimated moments to approximate the null distribution of *Q* by a gamma distribution and then apply that distribution in a homogeneity test based on *Q* (to be denoted *Q*_*γ*_) that is substantially more accurate than the use of the chi-square distribution. We also compare the accuracy and power of this test with those of other homogeneity tests, such as that of Breslow and Day [11]. Briefly, both the accuracy and the power of our test are comparable to those of the Breslow-Day test (see Sections“Accuracy of the level of the homogeneity test” and “Power of the homogeneity test)”.

After introducing notation and the main assumptions in Section “Notation and assumptions”, we proceed to our study of the moments of *Q* for log odds ratios in Section “The mean and variance of *Q*” and to their estimation in Section “Estimating the moments and distribution of ***Q***_***LOR***_”. Results of our simulations of the achieved level and power of the standard *Q* test, the Breslow-Day test and the proposed improved test of homogeneity based on *Q*_*γ*_ are given in Sections “Accuracy of the level of the homogeneity test” and “Power of the homogeneity test”. Section “Example: a meta-analysis of Stead et al. (2013)” contains an example from the medical literature to illustrate our results and to compare them to other tests. Section “Conclusions” contains a discussion and summary of our conclusions. We provide information on the design of our simulations in the Appendix; and more results of the simulations for various sample sizes, including unbalanced designs and unequal effects, are contained in the accompanying ‘Further Appendices’, together with additional information about the derivation of our procedures. Our R program for calculation of the *Q*_*γ*_ test of homogeneity can be downloaded from the Journal website.

## Methods

### Notation and assumptions

We assume that there are *K* studies each with two arms, which we call ‘treatment’ and ‘control’ and use the subscripts *T* and *C*. The sizes of the arms of the *i*th study are *n*_*Ti*_ and *n*_*Ci*_; let *N*_*i*_=*n*_*Ti*_+*n*_*Ci*_ and let *q*_*i*_=*n*_*Ci*_/*N*_*i*_. Data in the arms have binomial distributions with probabilities *p*_*Ti*_ and *p*_*Ci*_. The effect of interest is the logarithm of the odds ratio *θ*_*i*_= log[*p*_*Ti*_/(1−*p*_*Ti*_)]− log[*p*_*Ci*_/(1−*p*_*Ci*_)]. The null hypothesis to be tested is the equality of the odds ratios (or equivalently their logarithms) across the several studies, i.e., *θ*_1_=⋯=*θ*_*K*_:=*θ*.

To estimate *θ*_*i*_, we follow Gart, Pettigrew and Thomas [12] who showed that if *x* successes occur from the binomial distribution *Bin*(*n*;*p*), then among the estimators of log[*p*/(1−*p*)] given by *L*_*a*_(*x*)= log[(*x*+*a*)/(*n*−*x*+*a*)], the estimator with *a*=1/2 has minimum asymptotic bias; and indeed, this is the only choice of *a* for which all terms for the bias in the expansion of *L*_*a*_(*x*) having order *O*(1/*n*) vanish. Gart et al. [12] also show that

(1) $$ \text{Var}[L_{1/2}]=\frac{1}{np(1-p)}+\frac{(1+2p)^{2}}{2n^{2}p^{2}(1-p)^{2}}+O\left(1/n^{3}\right)   $$

and suggest the use of the following unbiased estimator of the variance: (*x*+1/2)^−1^+(*n*−*x*+1/2)^−1^. Accordingly, if *x*_*i*_ and *y*_*i*_ are the number of successes in the treatment and control arms of the *i*th study, we estimate *θ*_*i*_ by $\widehat {\theta }_{i}= L_{1/2}(x_{i})-L_{1/2}(y_{i})$. We estimate the variance of $\widehat {\theta }_{i}$ by

(2) $$ \begin{aligned} \widehat{\text{Var}}[\widehat{\theta}_{i}]=&\,\frac{1}{x_{i}+1/2}+ \frac{1}{n_{Ti}-x_{i}+1/2}+\frac{1}{y_{i}+1/2}\\&+ \frac{1}{n_{Ci}-y_{i}+1/2}. \end{aligned}   $$

A weight *w*_*i*_ is assigned to the *i*th study as the inverse of the variance of $\widehat {\theta }_{i}$, and the weight is estimated by $\widehat {w}_{i}=\widehat {\text {Var}}[\widehat {\theta }_{i}]^{-1}$. The weighted average of the log odds ratio effects is given by $\widehat {\theta }_{w}=\sum _{i}\widehat {w}_{i}\widehat {\theta }_{i}/\sum _{i}\widehat {w}_{i}$. Then Cochran’s *Q* statistic is defined as the weighted sum of the squared deviations of the individual effects from the average; that is,

(3) $$ Q=\sum_{i=1}^{K}\widehat{w}_{i}(\widehat{\theta}_{i}-\widehat{\theta}_{w})^{2}.   $$

The “standard” version of the *Q* statistic, denoted *Q*_*stand*_ does not add 1/2 to the number of events in both arms when calculating log-odds unless this is required to define their variances.

The distribution of *Q* under the null hypothesis of equality of the effects *θ*_*i*_ depends on the value of the common effect *θ*, the number of studies *K* and the sample sizes *n*_*Ti*_ and *n*_*Ci*_. However additional information is needed to specify a unique distribution for *Q*. For example, the common effect *θ*=0 (that is, the probabilities for the treatment and control arms are equal), could arise with all probabilities equal to 1/2 (in both arms of all studies) or with some of the studies having probabilities of 1/4 in both arms and others having probabilities of 1/3 in both arms. To uniquely specify a distribution for *Q*, we need to introduce a ‘nuisance’ parameter *ζ*_*i*_ for each study. It is convenient to take *ζ*_*i*_= log[*p*_*Ci*_/(1−*p*_*Ci*_)] to be the log odds for the control arm of the *i*th study and to estimate it as described above, i.e., $\widehat {\zeta }_{i}=L_{1/2}(y_{i})$.

### The mean and variance of *Q*

The *Q* statistic has long been known to behave asymptotically, as the sample sizes become large, as a chi-square distributed random variable with mean *K*−1 and variance, which is necessarily twice the mean, 2(*K*−1). However, the choice of effect (e.g., log odds ratio, sample mean, standardized mean difference) has a substantial impact on the distribution of *Q* for small to moderate sample sizes, which in turn affects the use of *Q* as a statistic for a test of homogeneity. For this section, we shall use the notation *Q*_*SM*_ for *Q* when the effect is a normally distributed sample mean and *Q*_*LOR*_ when the effect is the logarithm of the odds ratio.

Assuming that the data from the studies are distributed $N(\mu,{\sigma _{i}^{2}})$, Welch [3] and James [4] first studied the moments of *Q*_*SM*_ under the null hypothesis of homogeneity; using the normality properties, they calculated asymptotic expansions for the mean and variance of *Q*_*SM*_, and Welch matched these moments to those of a re-scaled F-distribution to create a homogeneity test now known as the Welch test. It is useful, for comparison with *Q*_*LOR*_, to examine Welch’s mean and variance for *Q*_*SM*_. Omitting terms of order $1/{n_{i}^{2}}$ and smaller, Welch found

(4) $$\begin{array}{@{}rcl@{}} \text{E}[Q_{SM}]=(K-1)+ 2\sum\frac{1-1/\left(W{\sigma_{i}^{2}}\right)}{n_{i}-1} \end{array} $$

(5) $$\begin{array}{@{}rcl@{}}  \text{Var}[Q_{SM}] = 2(K-1)+14\sum\frac{1-1/\left(W{\sigma_{i}^{2}}\right)}{n_{i}-1} \end{array} $$

where *W* is the sum of the “theoretical” weights $n_{i}/{\sigma _{i}^{2}}$. Notice the following facts about these moments. 1) They converge to the chi-square moments as the sample sizes increase. 2) Both moments are larger than the corresponding chi-square moments. We shall call the difference between the moments of *Q* and the corresponding chi-square moments: ‘corrections’. 3) The variance is more than twice the mean. 4) The moments depend on the nuisance parameters ${\sigma _{i}^{2}}$, which are estimated independently of the effects of interest (the sample means).

Based on a combination of theoretical expansions and extensive simulations, we have determined that, when the effect entering into the definition of *Q* is the log odds ratio, the mean and variance of *Q*_*LOR*_ (under the null hypothesis of equal odds ratios) have the following properties. 1) They each converge to the corresponding chi-square moments of *K*−1 and 2(*K*−1) as the sample sizes increase. 2) Both moments are less than the corresponding chi-square moments. That is, the ‘corrections’ are negative rather than positive as for *Q*_*SM*_. 3) The variance is not only less than the chi-square variance, it is less than twice the mean. 4) The moments depend on nuisance parameters, which are not independent of the effects.

**Figure 1:**
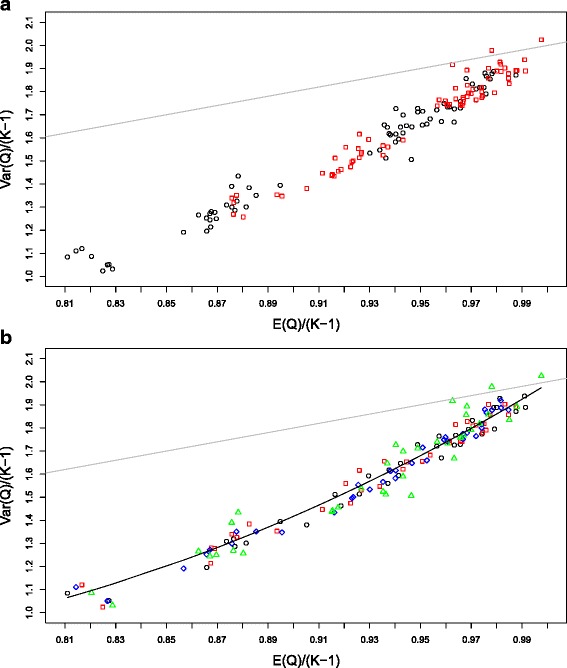
Variance vs mean of *Q*. This scatter plot of Var[*Q*]/(*K*−1) vs. E[*Q*]/(*K*−1) contains the results of simulations of the moments of *Q*_*LOR*_ for the 144 configurations of parameters: *K*= 5, 10, 20, 40; *N*= 90, 150, divided equally into the two arms; log odds ratios: 0, 0.5, 1, 1.5, 2, 3; and control probabilities: 0.1, 0.2, 0.4. The studies in each simulation all have the same parameters. The simulations for each configuration were replicated 10,000 times. The grey reference line (Var[*Q*]=2E[*Q*]) indicates the relation that would be expected if *Q* followed a chi-square distribution. (**a**): *N*=90 black and *N*=150 red. (**b**): *K*=5 (black), *K*=10 (red), *K*=20 (blue) and *K*=40 (green). The black curve corresponds to the fitted quadratic equation Var[*Q*_*LOR*_]/(*K*−1)=4.74−12.17E[*Q*_*LOR*_]/(*K*−1)+9.42[E[*Q*_*LOR*_]/(*K*−1)]^2^.

The two plots of Figure 1 show the relation of the variance of *Q*_*LOR*_ to its mean for a representative set of simulations. (See Appendix Appendix A: Information about the simulations for a complete description of the simulations conducted). The two plots have identical data, but the points are colored according to the value of *N* in the left plot and according to the value of *K* in the right plot. The mean and variance of *Q*_*LOR*_ have been divided by *K*−1 in order to place the data on the same scale. The main message of the right plot (and a key finding of our simulations) is that this re-scaling is effective—the different values of *K* (5, 10, 20 and 40) are fairly uniformly distributed throughout the plot, indicating that after this re-scaling the moments of *Q*_*LOR*_ have little dependence on the number of studies.

In the plots, we see that the mean of *Q*_*LOR*_ is less than *K*−1, that the variance of *Q*_*LOR*_ is less than 2(*K*−1), and that the variance is less than twice the mean. We also see in the left plot that the departure of the mean and variance from the chi-square values of *K*−1 and 2(*K*−1) (that is, the ‘corrections’) are greater for the study size *N*=90 (i.e., 45 in each arm) than for the study size *N*=150. It is not evident from the graphs, but the ‘corrections’ needed are also greater when the binomial probabilities *p*_*T*_ and *p*_*C*_ are more distant from the central value of 1/2.

### Estimating the moments and distribution of ***Q***_***LOR***_

In this section, we outline a method for estimating the mean and variance of *Q*_*LOR*_. The method involves fairly complicated formulas, but in the Appendix we provide more details and a link to a program in R for carrying out the calculations.

Kulinskaya et al. [10] presented a very general expansion for the mean of *Q* for arbitrary effect measures in terms of the first four central moments of the effect and nuisance parameters as well as the weight function expressed in terms of these parameters.

Necessary formulas for the application of this expansion to the first moment of *Q*_*LOR*_ can be found in B.2 Information about formulas for mean and variance of *Q*_*LOR*_. The resulting expansion provides an approximation to the mean of *Q*_*LOR*_, which we will denote E_*th*_[*Q*_*LOR*_] where the subscript ‘*th*’ indicates that this expectation is entirely theoretical. It depends on the number of studies *K*, the sample sizes of the separate arms of the studies, the estimated values of the nuisance parameters $\widehat {\zeta }_{i}$, the values of the estimated weights and the estimated value of the effect $\widehat {\theta }$ under the null hypothesis.

When we compared E_*th*_[*Q*_*LOR*_] with the simulated values for the mean of *Q*_*LOR*_, we found that it does an excellent job of identifying the situations where ‘corrections’ are needed to the chi-square moment, but that it over-estimates the size of the ‘correction’ by a constant percentage of slightly more than 1/3 (*R*^2^=97.0*%*). More precisely, denoting the mean of *Q*_*LOR*_ by E[*Q*_*LOR*_], we have the relation

(6) $$ (K-1)-\text{E}[Q_{LOR}]= 0.687[(K-1)-\text{E}_{th}[Q_{LOR}]].   $$

Although this equation is based partly on theoretical calculations and partly on the results of simulations (the “0.687” factor), we note that after deciding on the use of the “0.687” factor we conducted new simulations to verify that it was not just a random consequence of the original simulations. More details on our simulations for this formula can be found in B.1 Additional graphs for accuracy of level and for power.

Kulinskaya et al. [10] also deduced a very general theoretical expansion for the second moment of *Q*, but when we applied this expansion to *Q*_*LOR*_ and compared it to our simulations, we found that the expansion is much too inaccurate to be of any use. We conjecture that this inaccuracy is due to non-uniform convergence of the expansions with respect to both the number of studies *K* and the values of the binomial parameters. Accordingly we have chosen to estimate the variance of *Q*_*LOR*_ using a quadratic regression formula from our simulations, as seen in Figure 1, but using more complete data than shown in those plots. As in the regression for the mean of *Q*_*LOR*_ we fitted a formula for the variance and then checked it against additional simulations (See the B.2 Information about formulas for mean and variance of *Q*_*LOR*_ for more details on our procedures). Our formula for estimating Var[*Q*_*LOR*_] is

(7) $$ \begin{aligned} \text{Var}[Q_{LOR}]=&\, 4.74(K-1) -12.17 \text{E}[Q_{LOR}]\\ &+9.42\text{E}[Q_{LOR}]^{2}/(K-1) \end{aligned}   $$

The quadratic regression fit, using 487 of our more than 1400 simulations, had an *R*^2^ value of 98.5%. In using this equation, we first need to calculate E[*Q*_*LOR*_] using Equation 6. This quadratic regression is depicted by the black curve on the plot (b) of Figure 1.

Although we do not have a theoretical justification for using a quadratic relation between the mean and variance of *Q*, such a functional relation between the mean and the variance of *Q* is often found under various conditions. For examples, in the asymptotic chi-square distribution of *Q*, the variance (twice the mean) is a linear function of the mean; and in the normally distributed sample mean situation of Equations (4) and (5), a little algebra shows that again the variance is a linear function of the mean. Further, in a common one-way random effects model, Biggerstaff and Tweedie [13] show that the variance of *Q* is a quadratic function of the mean.

Our simulations show that the family of gamma distributions fits the distribution of *Q*_*LOR*_ quite well. By matching the mean and variance of *Q*_*LOR*_ with the mean and variance of a gamma distribution, we arrive at an approximation for the distribution of *Q*_*LOR*_ which can be used to conduct a test of homogeneity for the equality of log odds ratios using *Q*_*LOR*_ as the test statistic. (The shape parameter *α* of the gamma distribution is estimated by *α*=E[*Q*_*LOR*_]^2^/Var[*Q*_*LOR*_], and the scale parameter *β* is estimated by *β*=Var[*Q*_*LOR*_]/E[*Q*_*LOR*_].) The accuracy of this test statistic and a comparison with other test statistics are discussed in the next section.

## Results and discussion

### Accuracy of the level of the homogeneity test

In this section we present the results of extensive simulations designed to analyze the accuracy of the levels of the test of homogeneity of log odds ratios using the *Q* statistic together with the gamma distribution estimated from the data by the methods of Section “Estimating the moments and distribution of ***Q***_***LOR***_”. We denote this test by *Q*_*γ*_. The use of simulations to determine the accuracy of various different tests of homogeneity of log odds ratios has often been discussed in the literature. See, for example, Schmidt et al. [14], Bhaumik et al. [15], Bagheri et al. [16], Lui and Chang [17], Gavaghan et al. [18], Reis et al. [19], Paul and Donner [20,21], and Jones et al. [22]. Our simulations included comparisons with some of the tests proposed by these authors. The comparisons of ours confirmed (as several of the above authors also discovered) that the Breslow-Day [11] (denoted by BD) is often the best available among the previously considered tests.

The Breslow-Day test for homogeneity of odds-ratios is based on the statistic

$$X^{2}_{BD}=\sum_{j=1}^{K}\frac{(x_{j}-X_{j}(\hat{\psi}))^{2}}{\text{Var}(x_{j}|\hat{\psi})}, $$

where $x_{j},\;X_{j}(\hat {\psi })$ and $\text {Var}(x_{j}|\hat {\psi })$ denote the observed number, the expected number and the asymptotic variance of the number of events in the treatment arm of the *j*th study given the overall Mantel-Haenszel odds ratio $\hat {\psi }$, respectively. Its distribution is approximated by the *χ*^2^ distribution with *K*−1 degrees of freedom. We found that using the Tarone [23] correction to the Breslow-Day test had such small differences from BD that the two were virtually equivalent. In addition to the BD and Tarone tests, we simulated proposals by Lui and Chang [17] for testing the homogeneity of log odds ratios based on the normal approximation to the distribution of the *z*-, square-root and log-transformed *Q*_*stand*_ statistic. The log-transformation was also suggested by Bhaumik et al. [15]. We do not report these results due to our conclusion that none were superior to BD. Accordingly, in our comparative graphs below, we compare our *Q*_*γ*_ test with BD and with the commonly used test (denoted $Q_{\chi ^{2}}\phantom {\dot {i}\!}$), which uses the standard statistic *Q*_*stand*_ (calculated without adding 1/2 to the numbers of events when calculating log-odds) together with the chi-square distribution.

Our simulations for testing the null hypothesis of equal odds ratios (all conducted subsequent to the adoption of the regressions of Equations 6 and 7) are of two types. For the first type, the parameters of all studies are identical; these simulations include the following parameters: number of studies *K*= 5, 10, 20 and 40; total study sizes *N*= 90, 150, and 210; proportion of the study size in the control arm *q*= 1/3, 1/2, 2/3; null hypothesis value of the log odds ratio *θ*= 0, 0.5, 1, 1.5, 2, and 3; and the log odds of the control arm *ζ*= –2.2 (*p*_*C*_=0.1), –1.4 (*p*_*C*_=0.2) and –0.4 (*p*_*C*_=0.4). The second type of simulation fixes the null hypothesis values of equal log odds ratio at *θ*= 0, 0.5, 1, 1.5, 2, and 3, but the individual studies are quite heterogeneous concerning all other parameters. For example, for a null value of *θ*=0.5 and *K*=5 studies, one configuration with an average study size of 150 has different sample sizes of 96, 108, 114, 120, 312, each divided equally between the two arms (*q*=1/2) and different control arm probabilities *p*_*C*_ of 0.15, 0.3, 0.45, 0.6, and 0.75; note that the condition *θ*=0.5 when used with the five different control arm probabilities then uniquely specifies five probabilities *p*_*T*_ for the treatment arms. A complete description of the heterogeneous simulations can be found in Appendix Appendix A: Information about the simulations. When *K*= 5, 10 and 20, all simulations were replicated 10,000 times and thus approximate 95% confidence intervals for the achieved levels are ±0.004; but when *K*=40, the simulations were replicated only 1,000 times, giving approximate 95% confidence intervals for the levels of ±0.014.

**Figure 2:**
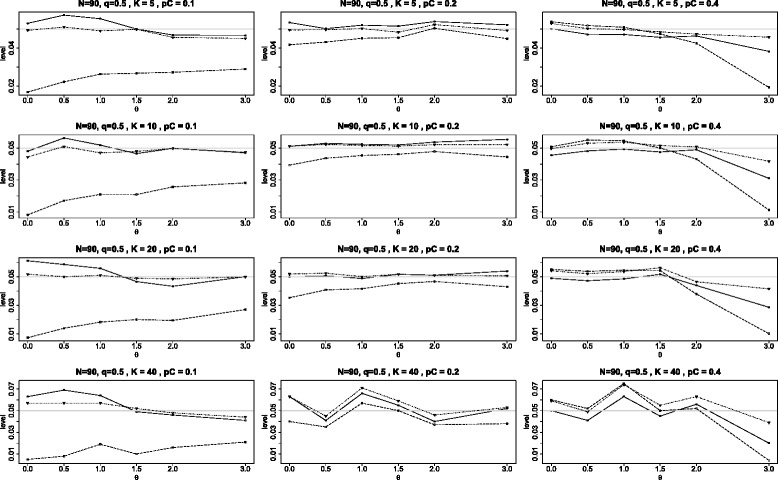
Achieved levels for homogeneous studies, *N*=90. Comparison of achieved levels, at the nominal level of 0.05, for the three tests *Q*_*γ*_ (solid line), BD (dot-dash), and $Q_{{\chi }^{2}}$ (dash) plotted against the log odds ratio *θ*. Here all studies have the same parameters: 90 subjects in each study with equal arms of 45 each (*N*=90 and *q*=1/2).

The first panel of graphs (see Figure 2) shows the achieved levels, at the nominal level of 0.05, for the three tests plotted against the different null values of *θ* in the range 0 to 3 under the configuration in which all *K* studies have identical parameters and the study sizes are *N*=90 with the subjects split equally between the two arms (*q*=1/2). The twelve graphs in the panel use *K*= 5, 10, 20 and 40; and *p*_*C*_= 0.1, 0.2, and 0.4. Note that the achieved levels for both BD and *Q*_*γ*_ are almost always in the range 0.04 to 0.06, with BD slightly better for many situations, but with *Q*_*γ*_ occasionally slightly better. The test $Q_{\chi ^{2}}\phantom {\dot {i}\!}$ is almost always inferior; and when *p*_*C*_=0.1, it is much too conservative (not rejecting the null hypothesis frequently enough); indeed, when *θ*=0, the achieved levels for $Q_{\chi ^{2}}\phantom {\dot {i}\!}$ are less than 0.01. In the four right graphs, when *p*_*C*_=0.4, we see that all three tests perform well when 0≤*θ*≤1.5; these parameters correspond to *p*_*T*_= 0.4, 0.52, 0.64 and 0.75. We also note that in the fairly extreme situation when *θ*=3 and *p*_*C*_=0.4 (and hence *p*_*T*_=0.93) the quality of all the tests worsens, however BD performs best here and $Q_{\chi ^{2}}\phantom {\dot {i}\!}$ performs very badly.

These results for the test $Q_{\chi ^{2}}\phantom {\dot {i}\!}$ are perhaps more easily understood when expressed in terms of the natural parameters, the binomial probabilities *p*_*C*_ and *p*_*T*_, rather than the log odds ratio *θ*. We see that $Q_{\chi ^{2}}\phantom {\dot {i}\!}$ is extremely conservative whenever either binomial parameter is far from the central values of 0.5, but that its performance is reasonable when the binomial parameters are relatively close to the central values of 0.5.

Figure 2 is representative of a number of additional panels of graphs for equal study sizes which can be found in B.1 Additional graphs for accuracy of level and for power, Figures 9 and 10. There we have included panels of graphs first for balanced arms with study sizes of 150 and 210. These panels are quite similar to the one presented in Figure 2 except that all levels become closer to the nominal level of 0.05 as the study size increases from 90 to 150 to 210. This behavior is consistent with the known fact that the tests are asymptotically correct as the study sizes tend to *∞*. However, we note that even when *N*=210, the test $Q_{\chi ^{2}}\phantom {\dot {i}\!}$ is still quite conservative when *p*_*C*_=0.1.

B.1 Additional graphs for accuracy of level and for power contains two additional panels of graphs (Figures 11 and 12) which are analogous to the panel in Figure 2 except that the two arms of each study are unbalanced. In the first of these, all studies have twice the number of subjects in the treatment arm (*q*=1/3) and the second is reversed with all studies having twice the number of subjects in the control arm (*q*=2/3). The results are similar to those of Figure 2 with the following modified conclusions. When *q*=1/3 and *p*_*c*_=0.1, the $Q_{\chi ^{2}}\phantom {\dot {i}\!}$ test is particularly conservative, rejecting the null hypothesis less than 1% of the time, independent of the number of studies *K*. Generally both the BD test and the *Q*_*γ*_ tests are reasonably close to nominal level, but the BD test is mostly (but not always) somewhat better than the *Q*_*γ*_ test. When *θ*=3, all tests experience a decline in accuracy, with the BD test mostly superior.

**Figure 3:**
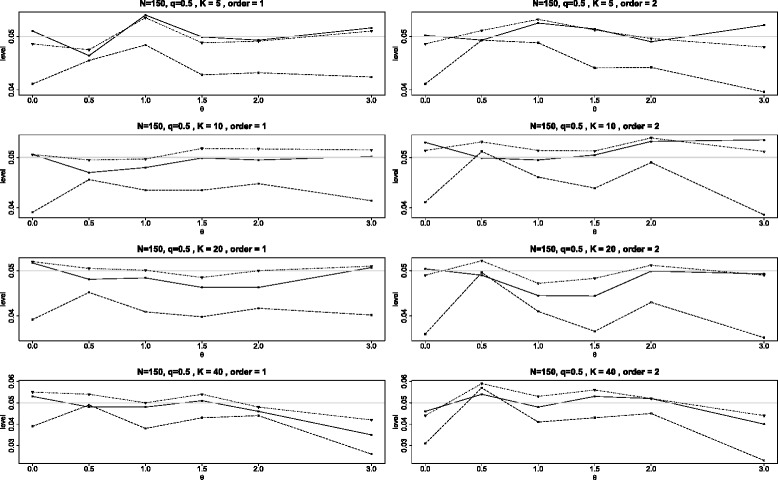
Achieved levels for heterogeneous studies, *N*=150. Comparison of achieved levels, at the nominal level of 0.05, for the three tests *Q*_*γ*_ (solid line), BD (dot-dash), and $Q_{{\chi }^{2}}$ (dash) plotted against the log odds ratio *θ* for heterogeneous studies. Here the studies have average size 150 divided equally between arms, but the study sizes and the binomial parameters vary for each study. In the left graphs, the smallest control probabilities are paired with the smallest study sizes. In the right graphs, the smallest control probabilities are paired with the largest study sizes.

Figure 3 is a typical example showing the achieved levels for one set of configurations in which all the studies are distinct. Here the studies are of average size 150. When *K*=5, the total study sizes are 96, 108, 114, 120, 312; in selecting these sizes, we have followed a suggestion of Sánchez-Meca and Marín-Martínez [24] who selected study sizes having the skewness 1.464, which they considered typical for meta-analyses in behavioral and health sciences. For a given *θ* the five studies had different values for the control arm and treatment arm probabilities (see Appendix for details). For *K*=10, 20 and 40, the parameters for *K*=5 were repeated 2, 4 and 8 times respectively. We see that BD and *Q*_*γ*_ are fairly close in outcome with achieved levels almost always between 0.045 and 0.055, while the levels for $Q_{\chi ^{2}}\phantom {\dot {i}\!}$ mostly cluster around 0.04. Note that the performance of $Q_{\chi ^{2}}\phantom {\dot {i}\!}$ is somewhat better than seen in Figure 2 for two reasons. First, the study sizes are larger (average of 150 rather than all having size 90); and second, because the binomial parameters vary among the different studies, many of them are closer to the central values of 0.5 where we have seen that the performance of the $Q_{\chi ^{2}}\phantom {\dot {i}\!}$ test improves.

It is worth noting that when we conducted simulations for the average sample size of 90 for the same scenario (so that the sample sizes were 36, 48, 54, 60, 252), we discovered that the Breslow-Day test does not perform well and may even not be defined for large numbers of studies *K* due to the sparsity of the data. This is the reason that, for comparative purposes, we use larger sample sizes in Figure 3 than used in Figure 2.

### Power of the homogeneity test

In this section we report on the results from our (limited) simulations of power of the three tests: the *Q*_*γ*_, BD and $Q_{\chi ^{2}}\phantom {\dot {i}\!}$ tests. Power comparisons are not really appropriate when the levels are inaccurate and differ across the tests. Unfortunately it is impossible to equalize the levels or adjust for the differences. Nevertheless we consider power comparisons at a nominal level of 0.05 to be important to inform the practice. We have performed simulations only for the case of *K* identical studies with balanced sample sizes (*q*=1/2). The values for the total study sizes *N*, the number of studies *K*, control arm probabilities *p*_*C*_ and the common log-odds ratio *θ* were identical to those used in simulating the levels for the identical studies given in Section “Accuracy of the level of the homogeneity test”. For each combination of *N*, *K*, *p*_*C*_, *θ*, according to the random effects model of meta-analysis, we simulated *K* within-studies log odds ratios *θ*_*i*_ from the *N*(*θ*,*τ*^2^) distribution for the values of the heterogeneity parameter *τ* from 0 to 0.9 in the increments of 0.1. Given the values of *p*_*C*_ and *θ*_*i*_, we next calculated the probabilities in the treatment groups *p*_*Ti*_ and simulated the numbers of the study outcomes from the binomial distributions Bin(*n*_*i*_,*p*_*C*_) and Bin(*n*_*i*_,*p*_*Ti*_) for *i*=1,⋯,*K*. All simulations were replicated 1000 times.

**Figure 4:**
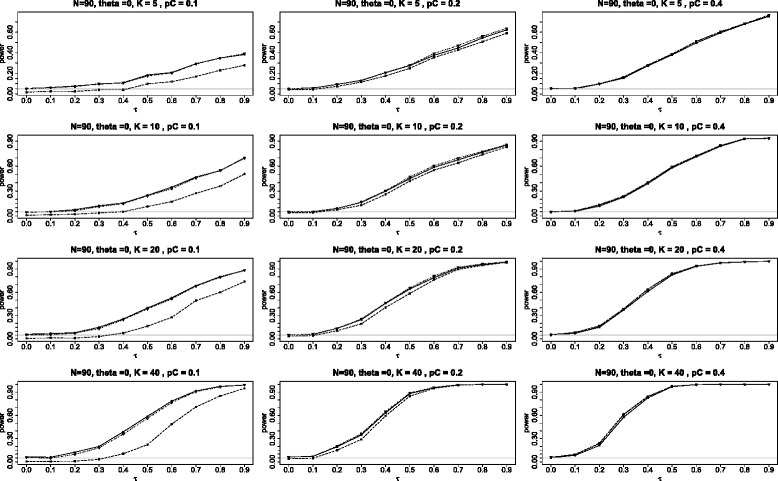
Power when the log odds ratio *θ*=0. Comparison of power for the three tests *Q*_*γ*_ (solid line), BD (dot-dash), and $Q_{{\chi }^{2}}$ (dash) plotted against *τ*, the square root of the random variance component *τ* ^2^. Here all studies have the parameters: 90 subjects in each study with equal arms of 45 each (*N*=90 and *q*=1/2) and the log odds ratio *θ*=0.

The first panel of graphs (see Figure 4) shows the power for the three tests when *θ*=0 plotted against the different values of heterogeneity parameter *τ* in the range 0 to 0.9 under the configuration in which all *K* studies have identical parameters, the study sizes are *N*=90 with the subjects split equally between the two arms (*q*=1/2). The twelve graphs in the panel use *K*= 5, 10, 20 and 40; and *p*_*C*_= 0.1, 0.2, and 0.4.

Note that the power for both BD and *Q*_*γ*_ are almost always higher than for $Q_{\chi ^{2}}\phantom {\dot {i}\!}$, with the difference being especially pronounced for *p*_*C*_=0.1. The inferiority of $Q_{\chi ^{2}}\phantom {\dot {i}\!}$ is due to its conservativeness noted in the Section “Accuracy of the level of the homogeneity test”. There is no clear-cut winner between the BD and the *Q*_*γ*_, with BD slightly better for some situations, but slightly worse for others. In the three right graphs, when *p*_*C*_=0.4, we see that all three tests perform equally well.

**Figure 5:**
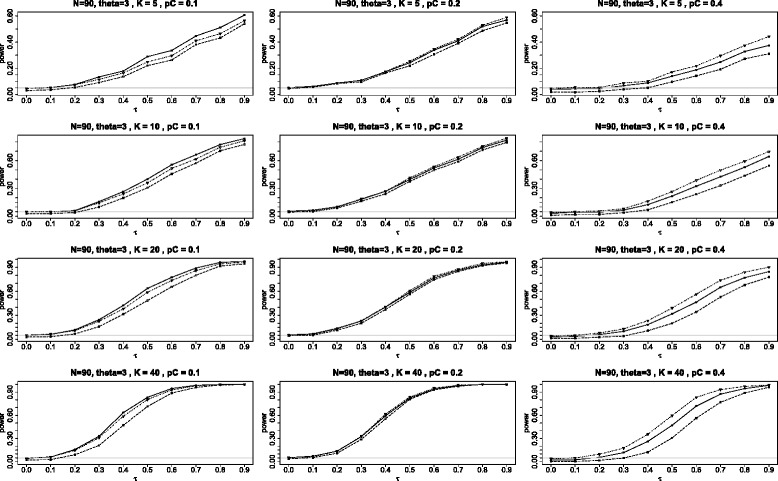
Power when the log odds ratio *θ*=3. Comparison of power for the three tests *Q*_*γ*_ (solid line), BD (dot-dash), and $Q_{{\chi }^{2}}$ (dash) plotted against *τ*, the square root of the random variance component *τ* ^2^. Here all studies have the parameters: 90 subjects in each study with equal arms of 45 each (*N*=90 and *q*=1/2) and the log odds ratio *θ*=3.

The second panel of graphs (see Figure 5) shows the power for the three tests when *θ*=3. The power of the $Q_{\chi ^{2}}\phantom {\dot {i}\!}$ test is still the lowest of the three tests. But here the power of the *Q*_*γ*_ test appears to be somewhat higher then for the BD when *p*_*C*_=0.1, about the same when *p*_*C*_=0.2, and noticeably lower in the extreme situation when *p*_*C*_=0.4. These differences in power between the BD and *Q*_*γ*_ tests are both the consequences of the fact that the *Q*_*γ*_ test is somewhat liberal for *p*_*C*_=0.1 and somewhat conservative for *p*_*C*_=0.4, as can be seen from Figure 2. The BD test is the closest to the nominal level in these circumstances.

### Example: a meta-analysis of Stead et al. (2013)

This section illustrates the theory of Sections “The mean and variance of *Q*” and “Estimating the moments and distribution of ***Q***_***LOR***_” and gives an indication of the improvement in accuracy of the homogeneity test. The calculations can be performed using our computer program (Additional files 1, 2 and 3).

**Figure 6:**
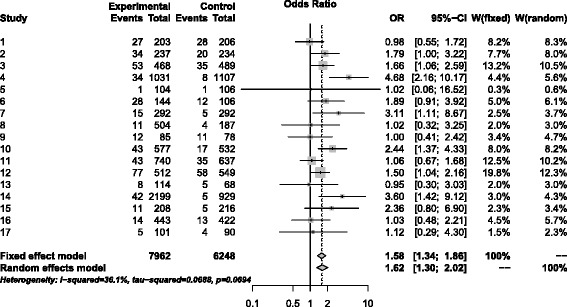
Forest plot of the meta-analysis by Stead et al. [25]. Forest plot of the meta-analysis by Stead et al. (2013) including 17 pre-2004 studies only, produced by the R package meta [26].

We use the data from the review by Stead et al. [25] of clinical trials on the use of physician advice for smoking cessation. Comparison 03.01.04 [25], p.65 considered the subgroup of interventions involving only one visit. We use odds ratio in our analysis below although relative risk was used in the original review. The first version of the review was published in 2001. Update 2, published in 2004, included 17 studies for this comparison. Summary data and the results from the standard analysis of these 17 trials are found in Figure 6, produced by the R package meta [26]. Note that meta does not add 1/2 to the number of events in calculation of the log-odds, and therefore calculates the standard statistic *Q*_*stand*_ for the test of homogeneity.

The value of Cochran’s *Q* statistic is 25.023. The standard chi-square approximation with 16 df yields the p-value of 0.069 for the test for homogeneity. The estimated mean E_*th*_[*Q*] of the null distribution of *Q* is 14.18 and the corrected mean using Equation 6 is E[*Q*]=14.75. The estimated variance calculated from Equation 7 is 24.43. The parameters of the approximating gamma distribution are *α*=8.90 and *β*=1.66. The p-value using this gamma distribution is 0.037. The Breslow-Day statistic value is 26.22 and the p-value is 0.051; the Tarone correction provides the same values to 4 decimal places. To evaluate the correctness of these p-values, we simulated one million values of *Q* from the fixed null distribution with each study having the null value *θ*_*w*_=1.58 for the odds ratio together with the original individual values for the control parameters *p*_*Ci*_. The conclusion, based on the empirical results, is that the p-value should be 0.0330. Thus for this example, the gamma distribution result is closest to that given by the simulations and the standard chi-square value is furthest.

The most current version of the review (Update 4) contains only one more trial by Unrod (2007) for this comparison. The values are event_*T*_=28, event_*C*_=18, *n*_*T*_=237, *n*_*C*_=228. With the addition of these data, the test of heterogeneity results in *Q*=25.023, and the p-value of 0.094 is obtained by the standard chi-square approximation with 17 df. Our method results in E_*th*_[*Q*]=15.14, and the corrected value E[*Q*]=15.72, Var[*Q*]=26.22, with the gamma distribution parameters *α*=9.43 and *β*=1.67. The p-value from the gamma approximation is 0.055. The BD test statistic is 26.22 and its p-value is 0.071; the Tarone correction, once more, results in the same values to 4 decimal places. Another set of one million simulations from the null distribution yielded the empirical p-value of 0.0497.

For the data in these two examples, the gamma approximation results in lower and more accurate p-values than the p-values of both the standard chi-square approximation and the Breslow-Day test. However, in our more extensive simulations there were cases in which the Breslow-Day test was superior. Note that this example has fairly low numbers of events (between 1% and 5% for many studies), which, as mentioned at the end of Section “Accuracy of the level of the homogeneity test”, is a situation where the Breslow-Day test may struggle.

**Figure 7:**
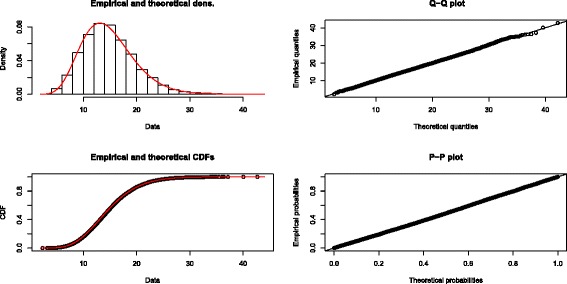
Quality of fit of the gamma approximation. Quality of fit of the gamma approximation (*α*=8.90 and *β*=1.66) to the empirical distribution of *Q* using the data of Stead et al. (2013) with 17 studies, produced by the R package fitdistrplus [30].

**Figure 8:**
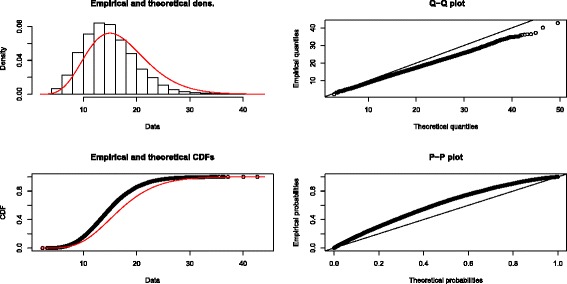
Quality of fit of the chi-square approximation. Quality of fit of the chi-square (16 degrees of freedom) approximation to the empirical distribution of *Q* using the data of Stead et al. (2013) with 17 studies, produced by the R package fitdistrplus [30].

Figures 7 and 8 provide a comparison which indicates the excellence of the fit of our gamma approximation to the entire distribution of *Q* and the poor fit of the chi-square approximation. Using the data of Stead et al. with 17 studies, we simulated 10,000 values of *Q* to provide an empirical distribution of *Q*. Figure 7 shows the fit of our estimated gamma distribution (*α*=8.90 and *β*=1.66). Note that the fit is quite good throughout the entire empirical distribution. On the other hand, Figure 8 shows that the empirical distribution of *Q* departs substantially from the chi-square distribution with 16 df, again throughout the entire distribution.

## Conclusions

Cochran’s *Q* statistic is a popular choice for conducting a homogeneity test in meta-analysis and in multi-center trials. However users must be cautious in referring *Q* to a chi-square distribution when the study sizes are small or moderate. Here we have studied the distribution of *Q* when the effects of interest are (the logarithms of) odds ratios between two arms of the individual studies. We have shown that the distribution of *Q* in these circumstances does not follow a chi-square distribution, especially if the binomial probability in at least one of the two arms is far from the central value of 0.5, say outside the interval [0.3,0.7]. Further, the convergence of the distribution of *Q* to the asymptotically correct chi-square distribution is relatively slow as the sizes of the studies increase.

The mean and variance of *Q* (when the effects are log odds ratios and under the null hypothesis of homogeneity) are often substantially less than the corresponding chi-square values. We have provided formulas for estimating these moments and have found that matching these moments to those of a gamma distribution provides a good fit to the distribution of *Q*. The use of this distribution for *Q* yields a reasonably good test of homogeneity (denoted *Q*_*γ*_) which is competitive with the well known Breslow-Day test both in accuracy of level and in power. However, this *Q*_*γ*_ test does not seem to be superior (either in accuracy of level or in power) to the Breslow-Day test. Accordingly we recommend that the simpler Breslow-Day test be used routinely for testing the homogeneity of odds ratios.

We note that when the data are very sparse, the Breslow-Day test does not perform well and may even not be defined. We have met this difficulty in our unequal simulations described in Section “Accuracy of the level of the homogeneity test”. The *Q*_*γ*_ test is always well defined and is recommended for use in such situations.

In our study of the moments of *Q* for log odds ratios, we found that the variance of *Q* can be well approximated by a function of the mean of *Q*. Thus when fitting a gamma distribution to *Q*, at least approximately, the resulting distribution comes from a one parameter sub-family of the gamma family of distributions. The chi-square distributions also form a one parameter sub-family of the gamma family, but our conclusion is that it is the wrong sub-family to apply to *Q*. Intuitively, one would expect that a two parameter family of distributions would be needed because two independent binomial parameters (*p*_*T*_ and *p*_*C*_) for each study enter into the definition of Q. Thus it would be of interest to have a theoretical explanation of this property of Q, but we have been unable to provide this explanation.

The *Q* statistic with its distribution approximated by the chi-square distribution is widely used not only for testing homogeneity, but perhaps a more widespread and more important use is its application to estimate the random variance component *τ*^2^ in a random effects model. Numerous moment-based estimation techniques, such as the very popular DerSimonian-Laird [6,27] and Mandel-Paule [28,29] methods use the first moment (*K*−1) and the chi-square percentiles applied to the distribution of *Q* to provide, respectively, point and interval estimation of *τ*^2^. The latter is achieved through ‘profiling’ the distribution of *Q*, i.e., inverting the *Q* test (see Viechtbauer [27]). From our previous work with Bjørkestøl on the homogeneity test for standardized mean differences [9] and for the risk differences [10], it is clear that the non-asymptotic distribution of *Q* strongly depends on the effect of interest. This conclusion is confirmed here for *Q* when the effects are log odds ratios. The use of the correct moments and improved approximations to the distribution of *Q* for the point and interval estimation of *τ*^2^ for a variety of different effect measures may provide greatly improved estimators, especially for small values of heterogeneity and will be the subject of our further work.

## Appendix

### Appendix A: Information about the simulations

All of our simulations for assessing the accuracy of the levels and the power of various homogeneity tests used *K* studies with *K*= 5, 10, 20 and 40. All simulations were replicated 10,000 times for *K*=5, 10 and 20, and (due to time considerations) only 1000 times for *K*=40, unless stated otherwise. The set of simulations with all studies having identical parameters were as follows: study size *N*= 90, 150 and 210; proportion of each study in the control arm *q* = 1/2, 1/3 and 2/3; log odds ratio (null hypothesis) *θ*= 0, 0.5, 1.0, 1.5, 2.0 and 3.0; and binomial probabilities in the control arm *p*_*C*_= 0.1, 0.2 and 0.4. It is easier and more intuitive to select values of *p*_*C*_ than to select values of the actual nuisance parameter *ζ*= log(*p*_*C*_)− log(1−*p*_*C*_).

For the simulations using unequal parameters among the various studies, the parameter choices can be described as follows. For *K*=5, we use three vectors of study sizes: <*N*>=<36,48,54,60,252>; <96,108,114,120,312>; and <163,173,178,184,352>. These three vectors have average study sizes 90, 150 and 210 respectively, which corresponds to the study sizes of the equal simulations. The null hypothesis values of the log odds ratio *θ* are 0, 0.5, 1.0, 1.5, 2 and 3. For each fixed value of *θ*, we chose five values of *p*_*C*_ with the goal of keeping *p*_*T*_ away from 1.0 (see below for these values). Denote the vector of these values of *p*_*C*_ by <*P*> and the vector of the same values but in reverse order by <∼*P*>. From *θ* and <*P*>, it is easy to calculate the corresponding values of *p*_*T*_; although these are not needed here, we include the approximate range of *p*_*T*_ for information purposes.

$$\begin{aligned} \theta &= 0 \qquad <P>= <0.1, 0.3, 0.5, 0.7, 0.9> \\&\hspace{45pt}\text{the range of}~~ p_{T} ~\text{is}~ [0.1, 0.9] \\ \theta &= 0.5 \quad\,\, <P>= < 0.15, 0.3, 0.45, 0.6, 0.75> \\&\hspace{45pt}\text{the range of}~~ p_{T} ~\text{is}~ [0.22, 0.83] \\ \theta &= 1.0 \quad\,\, <P>= < 0.1, 0.25, 0.4, 0.55, 0.7 >\\&\hspace{45pt}\text{the range of}~~ p_{T} ~\text{is}~ [0.23, 0.86] \\ \theta &= 1.5 \quad\,\,<P>= <0.1, 0.25, 0.4, 0.55, 0.7 > \\&\hspace{45pt}\text{the range of}~~ p_{T} ~\text{is}~ [0.33, 0.91] \\ \theta &= 2 \qquad\,<P>= <0.1, 0.2, 0.3, 0.4, 0.5 >\\&\hspace{45pt}\text{the range of}~~ p_{T} ~\text{is}~ [0.45, 0.88] \\ \theta &= 3 \qquad\, <P>= <0.1, 0.17, 0.24, 0.31, 0.38> \\&\hspace{45pt}{\text{~the range of}~~ p_{T} ~\text{is}~ [0.69, 0.92]} \end{aligned} $$

For *K*=5, we conducted simulations for each value of *θ* pairing the first value of <*N*> with the first value of <*P*>, etc. which we denote ‘order = 1’, and then we pair the first value of <*N*> with the first value of <∼*P*>, etc, which we denote ‘order = 2’. By reversing the orders, we first pair the largest study size with the largest binomial probability and then pair the largest study size with the smallest binomial probability. We used balanced studies for these simulations (i.e., *q*=1/2). For *K*=10, we repeat these pairings twice, and for *K*=20 and *K*=40 the vectors of study sizes and control arm probabilities are repeated 4 and 8 times respectively.

We conducted many additional simulations with unequal size studies, some with all control probabilities equal except for 20% of the studies which had different control probabilities, and some with one or more of the studies being unbalanced (*q*=1/3 and *q*=2/3). These simulations did not add substantial information to our conclusions, so they are not reported here.

For the power simulations we only considered the case of *K* studies with the above identical parameters (including the values of the common log odds ratio *θ*) and balanced sample sizes (*q*=1/2). For each combination of *N*, *K*, *p*_*C*_, *θ*, according to the random effects model of meta-analysis, we simulated *K* within-studies log odds ratios *θ*_*i*_ from the *N*(*θ*,*τ*^2^) distribution for the values of the heterogeneity parameter *τ* from 0 to 0.9 in the increments of 0.1. Given the values of *p*_*C*_ and *θ*_*i*_, we next calculated the probabilities in the treatment groups *p*_*Ti*_ and simulated the numbers of the study outcomes from the binomial distributions Bin(*n*_*i*_,*p*_*C*_) and Bin(*n*_*i*_,*p*_*Ti*_) for *i*=1,⋯,*K*. All simulations were replicated 1000 times.

## Appendix B

### B.1 Additional graphs for accuracy of level and for power

**Figure 9:**
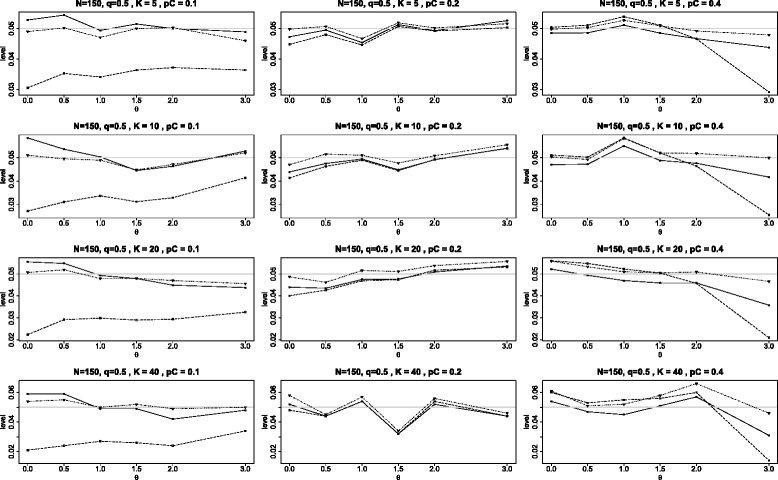
Achieved levels for homogeneous studies, *N*=150. Achieved levels for the three tests *Q*_*γ*_ (solid line), BD (dot-dash), and $Q_{{\chi }^{2}}$ (dash) plotted against the log odds ratio *θ*. Here all studies have the same parameters: 150 subjects in each study with equal arms of 75 each (*N*=150 and *q*=1/2).

**Figure 10:**
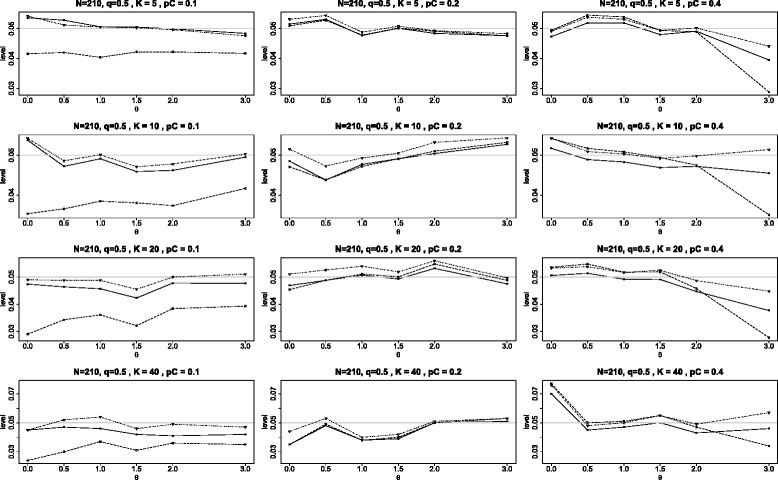
Achieved levels for homogeneous studies, *N*=210. Achieved levels for the three tests *Q*_*γ*_ (solid line), BD (dot-dash), and $Q_{{\chi }^{2}}$ (dash) plotted against the log odds ratio *θ*. Here all studies have the same parameters: 210 subjects in each study with equal arms of 105 each (*N*=210 and *q*=1/2).

The first two figures of this Appendix are similar to Figure 2 of the main article with the change being that the study sizes are 150 (instead of 90) in Figure 9 and 210 in Figure 10. These panels are quite similar to the one presented in Figure 2 except that all levels become closer to the nominal level of 0.05 as the study size increases from 90 to 150 to 210. This behavior is consistent with the known fact that the tests are asymptotically correct as the study sizes tend to *∞*. However, we note that even when *N*=210, the test $Q_{\chi ^{2}}\phantom {\dot {i}\!}$ is still quite conservative when *p*_*C*_=0.1.

**Figure 11:**
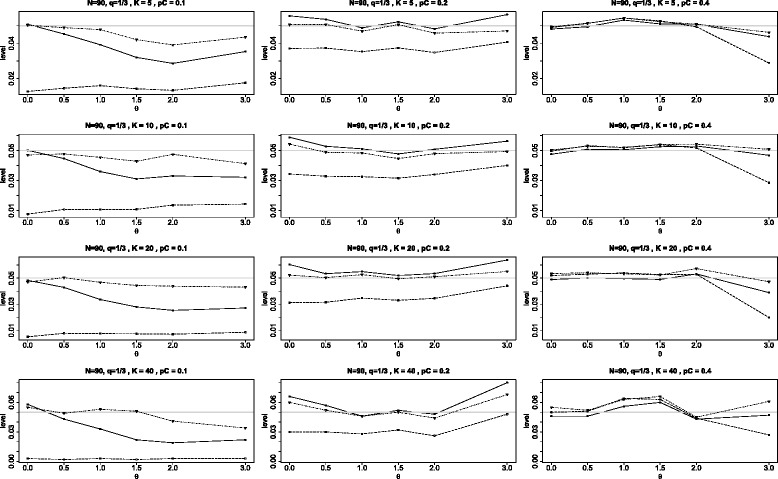
Achieved levels for homogeneous studies, *N*=90, *q*=1/3. Achieved levels for the three tests *Q*_*γ*_ (solid line), BD (dot-dash), and $Q_{{\chi }^{2}}$ (dash) plotted against the log odds ratio *θ*. Here all studies have the same parameters: 90 subjects in each study with unequal arms with 60 in the treatment arm (*N*=90 and *q*=1/3).

**Figure 12:**
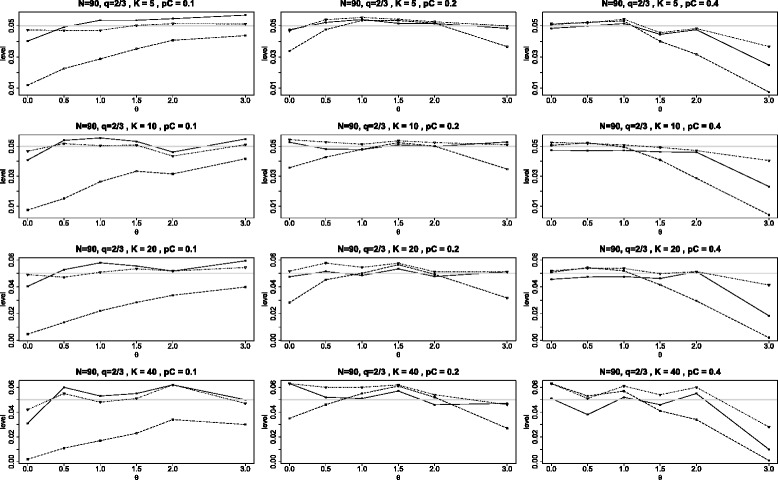
Achieved levels for homogeneous studies, *N*=90, *q*=2/3. Achieved levels for the three tests *Q*_*γ*_ (solid line), BD (dot-dash), and $Q_{{\chi }^{2}}$ (dash) plotted against the log odds ratio *θ*. Here all studies have the same parameters: 90 subjects in each study with unequal arms with 30 in the treatment arm (*N*=90 and *q*=2/3).

Figures 11 and 12 contain additional panels of graphs analogous to that in Figure 2 of the main article with the exception that the two arms of each study are unbalanced. In the first of these, all studies have twice the number of subjects in the treatment arm (*q*=1/3) and the second is reversed with all studies having twice the number of subjects in the control arm. The results are similar to those of Figure 2 with the following modified conclusions. When *q*=1/3 and *p*_*C*_=0.1, the $Q_{\chi ^{2}}\phantom {\dot {i}\!}$ test is particularly conservative, rejecting the null hypothesis less than 1% of the time, independent of the number of studies *K*. Generally both the BD test and the *Q*_*γ*_ test are reasonably close to nominal level, but the BD test is mostly (but not always) somewhat better than the *Q*_*γ*_ test. When *θ*=3, all tests experience a decline in accuracy, with the BD test mostly superior.

**Figure 13:**
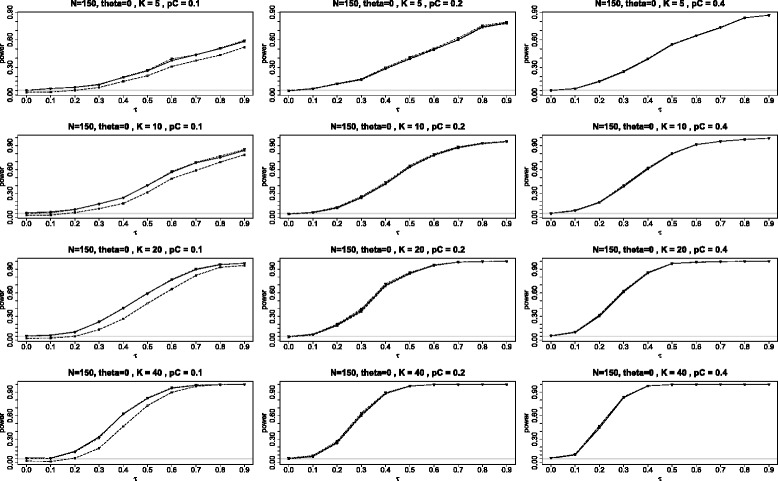
Power when the log odds ratio *θ*=0 and *N*=150. Power for the three tests *Q*_*γ*_ (solid line), BD (dot-dash), and $Q_{{\chi }^{2}}$ (dash) plotted against *τ*, the square root of the random effect variance. Here all studies have the parameters: 150 subjects in each study with equal arms of 75 each (*N*=150 and *q*=1/2) and the log odds ratio *θ*=0.

**Figure 14:**
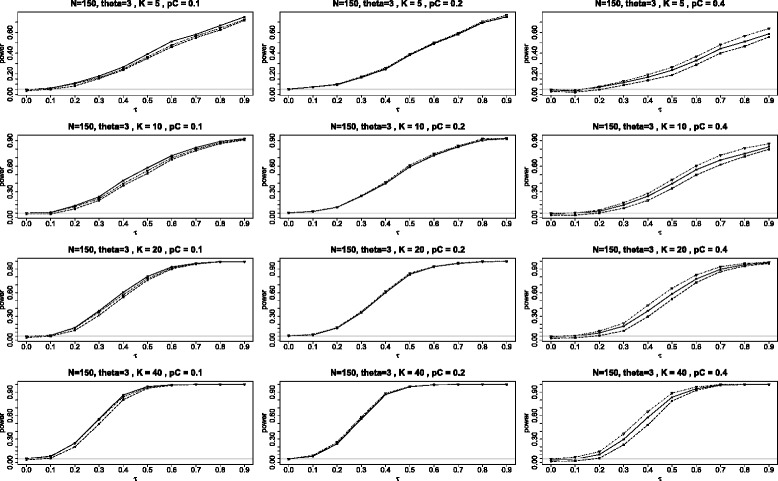
Power when the log odds ratio *θ*=3 and *N*=150. Power for the three tests *Q*_*γ*_ (solid line), BD (dot-dash), and $Q_{{\chi }^{2}}$ (dash) plotted against *τ*, the square root of the random effect variance. Here all studies have the parameters: 150 subjects in each study with equal arms of 75 each (*N*=150 and *q*=1/2) and the log odds ratio *θ*=3.

The final two figures in this appendix are analogous to Figures 4 and 5 in the main article, comparing the power of the three tests *Q*_*γ*_, BD and $Q_{\chi ^{2}}\phantom {\dot {i}\!}$ when the log odds ratio is 0 and 3 respectively. The panels here (Figures 13 and 14) differ in that the sample sizes have been increased from *N*=90 to *N*=150. Qualitatively the plots here are quite similar to those in the main article, with the main difference, as would be expected, being that the power when *N*=150 is somewhat greater than when *N*=90. As before, *Q*_*γ*_ and BD have similar power while $Q_{\chi ^{2}}\phantom {\dot {i}\!}$ is most inferior in the two cases: *θ*=0 and *p*_*C*_=0.1; and *θ*=3 and *p*_*C*_=0.4. These two cases share the property that one or both of the binomial probabilities is far from the central value of 0.5; in the first case, *p*_*C*_=*p*_*T*_=0.1 and in the second case, *p*_*T*_=0.93.

### B.2 Information about formulas for mean and variance of *Q*_*LOR*_

In this appendix we present additional information concerning the data and methods that entered into Equations 6 and 7 which provide formulas for estimating the mean and variance of *Q*_*LOR*_ under the null hypothesis of equal odds ratios. The data for Equation 6 include 648 parameter combinations in which all *K* studies had identical parameters. The parameters are: *K*= 5, 10, 20, 40; *N*= 90, 150, 210; *q*= 1/3, 1/2, 2/3; *p*_*C*_=0.1, 0.2, 0.4; and *θ*= 0, 0.5, 1, 1.5, 2, 3. The simulations for *K*=40 were replicated 1,000 times, and the other simulations were replicated 10,000 times.

**Figure 15:**
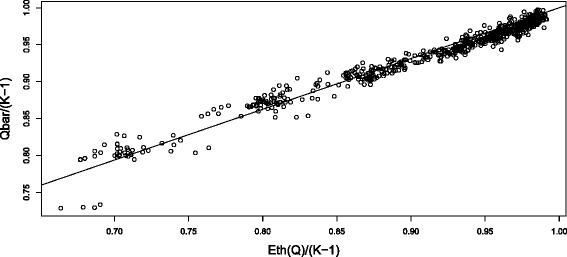
Fitted line plot for the first moment of *Q*. Fitted line plot of the relative first moment of *Q* based on studies with equal parameters. The horizontal coordinate is the first moment (divided by *K*−1) as estimated using a theoretical expansion, and the vertical coordinate is the first moment (divided by *K*−1) as found from the simulations.

For each combination of parameters, we calculated an estimate of the mean of *Q*_*LOR*_ (to be denoted simply *Q* in this section) using the theoretical expansion of Kulinskaya et al. [10]. We denote this quantity by E_*th*_[*Q*]. For each parameter combination, we also found the mean of *Q* from the simulations, which we denote by *Qbar*. These two quantities were then divided by *K*−1 to place the data on a scale common for all *K*. A scatter plot with a fitted line is found in Figure 15. Note that the fitted line (which has an *R*^2^ value of 97.0%) essentially goes through the point (1,1); the importance of the fitted line going through (1,1) is that both estimates agree when there is zero ‘correction’ from the re-scaled chi-square moment. Thus we subtracted 1 from both variables in Figure 15 and fit a regression through the origin, yielding a relation which we use to adjust the ‘corrections’ to the chi-square first moments *K*−1 which are given by the the expansion E_*th*_[*Q*]. This relation is found in Equation 6 of the main paper. (The four outliers in the lower left of Figure 15 belong to the extreme parameter values *θ*=3, *N*=90, *q*=2/3, *p*_*T*_=0.93, *p*_*C*_=0.4 and for the four values of *K*= 5, 10, 20 and 40; omitting them made very little difference in the regression, so they were included in the analysis). Simulations for all of the parameter configurations that entered into Equation 6 of the main paper were redone, and these new simulations were the ones used in analyzing the accuracy of our test *Q*_*γ*_.

**Figure 16:**
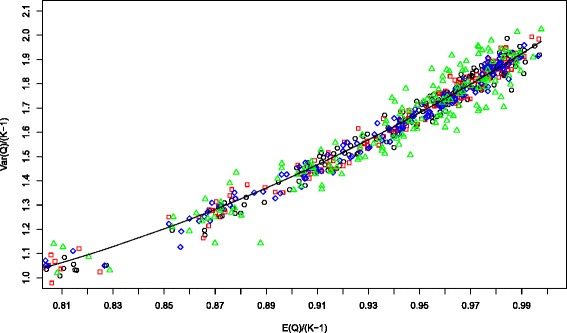
Quadratic fit between the variance and the mean of *Q*. Quadratic fit for the relation between the variance of *Q* and the mean of *Q*. The studies for differing values of *K* are depicted as: *K*=5 black circles; *K*=10 red squares; *K*=20 blue diamonds; *K*=40 green triangles.

To arrive at the relation in Equation 7, we used simulations for 486 parameter combinations in which all *K* studies have the same parameters: *K*= 5, 10, 20; *N*= 90, 150, 210; *q*= 1/3, 1/2, 2/3; *p*_*C*_ = 0.1, 0.2, 0.4; and *θ*= 0, 0.5, 1, 1.5, 2, 3, each replicated 10,000 times. For each parameter combination, let *Qbar* be the mean of the 10,000 values of *Q* and *VarQbar* be the variance of these 10,000 values of *Q*, and re-scale these values by dividing by *K*−1. Figure 16 contains a scatter plot of these data together with a quadratic function fit. The quadratic fit has an *R*^2^ value of 98.5%. We have used this regression in Equation 7 of the main article. We note again that simulations for all of the parameter configurations that entered into Equation 7 of the main paper were redone, and these new simulations were the ones used in analyzing the accuracy of our test *Q*_*γ*_.

### B.3 The general expansion for the first moment of ***Q*** applied to ***Q***_***LOR***_

The general expansion for the first moment of *Q* (denoted E_*th*_[*Q*] in Section “Estimating the moments and distribution of ***Q***_***LOR***_”) as found in Kulinskaya et al. [10] is reproduced at the end of this appendix. In the formulas below, we use the notation $\Theta _{i}=\widehat {\theta }_{i}-\theta _{i}$ and $Z_{i}=\widehat {\zeta }_{i}-\zeta _{i}$; also, we express the weight estimators as functions of the parameter estimators $\widehat {w}_{i}=f_{i}(\widehat {\theta }_{i}, \widehat {\zeta }_{i})$. The theoretical weights under the null hypothesis are then *w*_*i*_=*f*_*i*_(*θ*,*ζ*_*i*_). For the weights as defined in Equation 2 of the main artlcle, some algebra produces the formula for the weight function

(8) $$ \widehat{w}_{i}= f_{i}(\widehat{\theta}_{i}, \widehat{\zeta}_{i})=\left[\frac{(1+e^{\widehat{\theta}_{i}+\widehat{\zeta}_{i}})^{2}}{(n_{Ti}+1)e^{\widehat{\theta}_{i}+\widehat{\zeta}_{i}}} +\frac{(1+e^{\widehat{\zeta}_{i}})^{2}}{(n_{Ci}+1)e^{\widehat{\zeta}_{i}}}\right]^{-1}   $$

The formulas below require that the central moments of $\widehat {\theta }_{i}$ and $\widehat {\zeta }_{i}$ satisfy the following order conditions: $O(\text {E}[\Theta _{i}]) =1/{n_{i}^{2}}$, $O({\text {E}[\Theta _{i}^{2}}]) =1/n_{i}$, $O({\text {E}[\Theta _{i}^{3}}]) =1/{n_{i}^{2}}$ and $O({\text {E}[\Theta _{i}^{4}}]) =1/{n_{i}^{2}}$ and similar conditions for the central moments of $\widehat {\zeta }_{i}$. These order conditions for the specific case of the estimators of the log odds ratio (as defined in Section “Notation and assumptions”) follow from the work of Gart et al. [12]. However, instead of using the approximations for the central moments given by Gart et al., our R-program calculates these exactly.

The derivation of the expansion is a straightforward application of the delta method in which *Q* is first expanded in a multivariate Taylor series centered at the null hypothesis and then expectations are taken of the resulting expansion, keeping only those terms of order *O*(1) and *O*(1/*n*). For the Taylor expansion of *Q*, we consider *Q* as a function of the estimators of the effect and nuisance parameters as follows:

$$\begin{array}{@{}rcl@{}} Q&=&\sum_{i}\widehat{w}_{i}(\widehat{\theta}_{i}-\widehat{\theta}_{w})^{2}= Q[\widehat{\theta}_{1},\ldots,\widehat{\theta}_{K}, \widehat{w}_{1},\ldots,\widehat{w}_{K}]\\[-3pt] &=&Q[\widehat{\theta}_{1},\ldots,\widehat{\theta}_{K}, f_{1}(\widehat{\theta}_{1},\widehat{\zeta}_{1}),\ldots, f_{K}(\widehat{\theta}_{K},\widehat{\zeta}_{K})]. \end{array} $$

Under the null hypothesis all the effect parameter values are equal; that is, *θ*_1_=⋯=*θ*_*K*_, and we denote this common value by *θ*. The desired Taylor expansion of *Q* is centered at $\vec {\theta }:=(\theta, \ldots, \theta, \zeta _{1}, \ldots, \zeta _{K})$.

(9) $$ {\fontsize{9}{6} \begin{aligned} \text{E}_{th}[Q] =&\,\frac{1}{2}\sum_{i} \frac{\partial^2Q(\vec{\theta})}{\partial \widehat{\theta}_{i}^{2}} \text{E}\left[\Theta_{i}^{2}\right] + \frac{1}{6}\sum_{i} \frac{\partial^3Q(\vec{\theta})}{\partial\widehat{\theta}_{i}^{3}}\text{E}\left[\Theta_{i}^{3}\right]\\ &+ \frac{1}{2}\sum_{i} \frac{\partial^3 Q(\vec{\theta})}{\partial \widehat{\theta}_{i}^2 \partial\widehat{\zeta}_i}\text{E}\left[\Theta_{i}^2Z_{i}\right] + \frac{1}{24}\sum_{i}\frac{\partial^{4}Q(\vec{\theta})}{{\partial\widehat{\theta}_{i}^{4}}}\text{E}\left[\Theta_{i}^{4}\right]\\ &+ \frac{1}{6}\sum_{i} \frac{\partial^{4} Q(\vec{\theta})}{\partial {\widehat{\theta}_{i}^{3}} \partial\widehat{\zeta}_{i}}\text{E}\left[{\Theta_{i}^{3}}Z_{i}\right] +\frac{1}{4}\sum_{i} \frac{\partial^{4} Q(\vec{\theta})}{\partial {\widehat{\theta}_{i}^{2}} {\partial\widehat{\zeta}_{i}^{2}}}\text{E}\left[{\Theta_{i}^{2}}{Z_{i}^{2}}\right]\\ &+ \frac{1}{8}\sum_{i\neq j}\sum\frac{\partial^{4}Q(\vec{\theta})}{{\partial\widehat{\theta}_{i}^{2}}{\partial\widehat{\theta}_{j}^{2}}} \text{E}\left[{\Theta_{i}^{2}}\right]\text{E}\left[{\Theta_{j}^{2}}\right]\\ &+ \frac{1}{2}\sum_{i\neq j}\sum \frac{\partial^{4} Q(\vec{\theta})}{\partial \widehat{\theta}_{i} \partial \widehat{\zeta}_{i} \partial \widehat{\theta}_{j} \partial \widehat{\zeta}_{j}}\text{E}[\Theta_{i}Z_{i}]\text{E}[\Theta_{j} Z_{j}]\\ &+ \frac{1}{2}\sum_{i\neq j}\sum \frac{\partial^{4} Q(\vec{\theta})}{\partial {\widehat{\theta}_{i}^{2}} \partial \widehat{\theta}_{j}\partial \widehat{\zeta}_{j} }\text{E}\left[{\Theta_{i}^{2}}\right]\text{E}\left[\Theta_{j}Z_{j}\right]\\ &+\frac{1}{4}\sum_{i\neq j}\sum \frac{\partial^{4} Q(\vec{\theta})}{\partial {\widehat{\theta}_{i}^{2}}\partial {\widehat{\zeta}_{j}^{2}}}\text{E}\left[{\Theta_{i}^{2}}\right]\text{E}\left[Z_{j}^{2}\right]+ O\left(\frac{1}{n^{2}}\right) \end{aligned}}  $$

For the derivatives of *Q* needed in the above expansion. we use the notation $W=\sum _{i}w_{i}$ and *U*_*i*_=1−*w*_*i*_/*W* and evaluate all derivatives at the null hypothesis. All multi-index derivatives assume inequality of the indices *i* and *j*.

$${ \fontsize{8.6}{6}\begin{aligned} \frac{\partial^{2}Q(\vec{\theta})}{{\partial\widehat{\theta}_{i}^{2}}}&=2w_{i}U_{i}\\ \frac{\partial^{3}Q(\vec{\theta})}{{\partial\widehat{\theta}_{i}^{3}}}&=6{U_{i}^{2}}\frac{\partial f_{i}(\vec{\theta})}{\partial\widehat{\theta}_{i}}\\ \frac{\partial^{3}Q(\vec{\theta})}{{\partial\widehat{\theta}_{i}^{2}}\partial\widehat{\zeta}_{i}} &= 2{U_{i}^{2}}\frac{\partial f_{i}(\vec{\theta})}{\partial\widehat{\zeta}_{i}}\\ \frac{\partial^{4}Q(\vec{\theta})}{{\partial\widehat{\theta}_{i}^{4}}}&=12{U_{i}^{2}} \left[\frac{\partial^{2} f_{i}(\vec{\theta})}{{\partial\widehat{\theta}_{i}^{2}}}-\frac{2}{W}\left(\frac{\partial f_{i}(\vec{\theta})}{\partial\widehat{\theta}_{i}}\right)^{2}\right]\\ \frac{\partial^{4} Q(\vec{\theta})}{\partial {\widehat{\theta}_{i}^{3}} \partial\widehat{\zeta}_{i}} &= 6{U_{i}^{2}}\left[\frac{\partial^{2} f_{i}(\vec{\theta})}{\partial\widehat{\theta}_{i}\partial\widehat{\zeta}_{i}}-\frac{2}{W}\left(\frac{\partial f_{i}(\vec{\theta})}{\partial\widehat{\theta}_{i}}\right)\left(\frac{\partial f_{i}(\vec{\theta})}{\partial\widehat{\zeta}_{i}}\right)\right]\\ \frac{\partial^{4} Q(\vec{\theta})}{\partial {\widehat{\theta}_{i}^{2}} {\partial\widehat{\zeta}_{i}^{2}}}&= 2{U_{i}^{2}}\left[\frac{\partial^{2} f_{i}(\vec{\theta})}{{\partial\widehat{\zeta}_{i}^{2}}}-\frac{2}{W}\left(\frac{\partial f_{i}(\vec{\theta})}{\partial\widehat{\zeta}_{i}}\right)^{2}\right]\\ \frac{\partial^{4}Q(\vec{\theta})}{{\partial\theta_{i}^{2}}{\partial\theta_{j}^{2}}}&= \frac{-4}{W^{3}}\left[w_{j}^{2}\left(\frac{\partial f_{i}(\vec{\theta})}{\partial\widehat{\theta}_{i}}\right)^{2}+ {w_{i}^{2}}\left(\frac{\partial f_{j}(\vec{\theta})}{\partial\widehat{\theta}_{j}}\right)^{2}\right]\\ &+\frac{2}{W^{2}}\left[w_{j}^{2}\frac{\partial^{2} f_{i}(\vec{\theta})}{{\partial\widehat{\theta}_{i}^{2}}}+{w_{i}^{2}}\frac{\partial^{2} f_{j}(\vec{\theta})}{{\partial\widehat{\theta}_{j}^{2}}}\right] \\ &+\frac{8}{W^{2}}\left[w_{i}U_{j}+w_{j}U_{i}-W\right]\left(\frac{\partial f_{i}(\vec{\theta})}{\partial\widehat{\theta}_{i}}\right)\left(\frac{\partial f_{j}(\vec{\theta})}{\partial\widehat{\theta}_{j}}\right)\\ \frac{\partial^{4} Q(\vec{\theta})}{\partial \widehat{\theta}_{i} \partial \widehat{\zeta}_{i} \partial \widehat{\theta}_{j} \partial \widehat{\zeta}_{j}}&=\frac{2}{W^{2}}\left[w_{i}U_{j}+w_{j}U_{i}-W\right]\left(\frac{\partial f_{i}(\vec{\theta})}{\partial\widehat{\zeta}_{i}}\right)\left(\frac{\partial f_{j}(\vec{\theta})}{\partial\widehat{\zeta}_{j}}\right)\\ \frac{\partial^{4} Q(\vec{\theta})}{\partial {\widehat{\theta}_{i}^{2}} \partial \widehat{\theta}_{j}\partial \widehat{\zeta}_{j} }&= \frac{4}{W^{2}}\left[w_{i}U_{j}+w_{j}U_{i}-W\right]\left(\frac{\partial f_{i}(\vec{\theta})}{\partial\widehat{\theta}_{i}}\right)\left(\frac{\partial f_{j}(\vec{\theta})}{\partial\widehat{\zeta}_{j}}\right)\\ &+ \frac{2{w_{i}^{2}}}{W^{2}}\left(\frac{\partial^{2} f_{j}(\vec{\theta})}{\partial\widehat{\theta}_{j}\partial\widehat{\zeta}_{j}}\right)- \frac{4{w_{i}^{2}}}{W^{3}}\left(\frac{\partial f_{j}(\vec{\theta})}{\partial\widehat{\theta}_{j}}\right)\left(\frac{\partial f_{j}(\vec{\theta})}{\partial\widehat{\zeta}_{j}}\right)\\ \frac{\partial^{4} Q(\vec{\theta})}{\partial {\widehat{\theta}_{i}^{2}}\partial {\widehat{\zeta}_{j}^{2}}}&=\frac{2{w_{i}^{2}}}{W^{2}}\left(\frac{\partial^{2} f_{j}(\vec{\theta})}{{\partial\widehat{\zeta}_{j}^{2}}}\right)- \frac{4{w_{i}^{2}}}{W^{3}}\left(\frac{\partial f_{j}(\vec{\theta})}{\partial\widehat{\zeta}_{j}}\right)^{2} \end{aligned}} $$

## Additional files

Additional file 1
**R program for computing the homogeneity test** ***Q***_***γ***_ **.**

Additional file 2
**Additional R subroutines for computing the homogeneity test** ***Q***_***γ***_ **.**

Additional file 3
**Description of the R program for computing the homogeneity test** ***Q***_***γ***_ **.**

## Abbreviations

LOR: Log-odds ratio
BD: the Breslow-Day test

## References

[1] Cochran WG (1937). Problems arising in the analysis of a series of similar experiments. J R Stat Soc Suppl..

[2] Cochran WG (1954). The combination of estimates from different experiments. Biometrics.

[3] Welch BL (1951). On the comparison of several mean values: an alternative approach. Biometrika.

[4] James GS (1951). The comparison of several groups of observations when the ratios of the population variances are unknown. Biometrika.

[5] Woolf B (1955). On estimating the relation between blood group and disease. Ann Human Genet..

[6] DerSimonian R, Laird N (1986). Meta-analysis in clinical trials. Controlled Clin Trials.

[7] Higgins JPT, Thompson SG (2002). Quantifying heterogeneity in meta-analysis. Stat Med..

[8] Huedo-Medina TB, Sánchez-Meca J, Marín-Martínez F, Botella J (2006). Assessing heterogeneity in meta-analysis: Q statistic or *I*^2^ index?. Psychol Methods.

[9] Kulinskaya E, Dollinger MB, Bjørkestøl K (2011). Testing for homogeneity in meta-analysis I, The one parameter case: Standardized mean difference. Biometrics.

[10] Kulinskaya E, Dollinger MB, Bjørkestøl K (2011). On the moments of Cochran’s *Q* statistic under the null hypothesis, with application to the meta-analysis of risk difference. Res Synth Methods.

[11] Breslow NE, Day NE (1980). Statistical methods in cancer research. Int Agency Res Cancer.

[12] Gart JJ, Pettigrew HM, Thomas DG (1985). The effect of bias, variance estimation, skewness and kurtosis of the empirical logit on weighted least squares analysis. Biometrika.

[13] Biggerstaff BJ, Tweedie RL (1997). Incorporating variability in estimates of heterogeneity in the random effects model in meta-analysis. Stat Med..

[14] Schmidt AF, Groenwold RHH, Knol MJ, Hoes AW, Nielen M, Roes KCB (2014). Exploring interaction effects in small samples increases rates of false-positive and false-negative findings: results from a systematic review and simulations study. J Clin Epidemiol..

[15] Bhaumik DK, Amatya A, Normand S, Greenhouse J, Kaizar E, Neelon B (2012). Meta-analysis of rare binary adverse event data. J Am Stat Assoc..

[16] Bagheri Z, Ayatollahi SMT, Jafari P (2011). Comparison of three tests of homogeneity of odds ratios in multicenter trials with unequal sample sizes within and among centers. BMC Med Res Methodology.

[17] Lui K-J, Chang K-C (2009). Test homogeneity of odds ratio in a randomized clinical trial with noncompliance. J Biopharmaceutical Stat..

[18] Gavaghan DJ, Moore RA, McQuay HJ (2000). An evaluation of homogeneity tests in meta-analysis in pain using simulations of individual patient data. Pain.

[19] Reis IM, Hirji KF, Afifi AA (1999). Exact and asymptotic tests for homogeneity in several 2×2 tables. Stat Med..

[20] Paul SR, Donner A (1992). Small sample performance of tests of homogeneity of odds ratios in *K* 2×2 tables. Stat Med..

[21] Paul SR, Donner A (1989). A compararison of tests of homogeneity of odds ratios in *K* 2×2 tables. Stat Med..

[22] Jones MP, O’Gorman TW, Lemke JH, Woolson RF (1989). A Monte Carlo investigation of homogeneity tests of odds ratio under various sample size configurations. Biometrics.

[23] Tarone RE (1985). On heterogeneity tests based on efficient scores. Biometrika.

[24] Sánchez-Meca J, Marín-Martínez F (2000). Testing the significance of a common risk difference in meta-analysis. Comput Stat Data Anal..

[25] Stead LF, Buitrago D, Preciado N, Sanchez G, Hartmann-Boyce J, Lancaster T. Physician advice for smoking cessation. Cochrane Database Syst Rev. 2013; 5. Art. No.: CD000165. http://onlinelibrary.wiley.com/doi/10.1002/14651858.CD000165.pub4/full 10.1002/14651858.CD000165.pub4 PMC7064045 23728631

[26] Schwarzer G. meta, v.1.5-0. CRAN 2010. R package. Fixed and random effects meta-analysis. Functions for tests of bias, forest and funnel plot.

[27] Viechtbauer W (2007). Confidence intervals for the amount of heterogeneity in meta-analysis. Stat Med..

[28] Mandel J, Paule RC (1970). Interlaboratory evaluation of a material with unequal numbers of replicates. Anal Chem..

[29] DerSimonian R, Kacker R (2007). Random-effects model for meta-analysis of clinical trials: An update. Contemporary Clin Trials.

[30] Delignette-Muller ML, Dutang C. fitdistrplus, 1.0-4. CRAN 2015. R package. Help to Fit of a Parametric Distribution to Non-Censored or Censored Data.

