# Supplementary material for: An accurate test for homogeneity of odds ratios based on Cochran’s Q-statistic
Source: BMC Med Res Methodol. 2015 Jun 10;15:49. doi: 10.1186/s12874-015-0034-x (PMC4531442; doi:10.1186/s12874-015-0034-x)
Supplement: Additional file 3 — Description of the R program for computing the homogeneity test Q γ . [file 12874_2015_34_MOESM3_ESM.doc]

| new_QOR | R Documentation |
| --- | --- |

## Testing for homogeneity in the meta-analysis of odds ratios

### Description

This program provides an improved approximation to the distribution of Cochran’s Q statistic for the use in a test to detect heterogeneity in the meta-analysis of odds ratios (OR) for studies with binomial data. The program calculates the p-value from the Gamma approximation proposed in Kulinskaya and Dollinger (2015).

### Usage

### new_QOR(nT,nC,XT,XC)

### Arguments

| nT | Number of observations in the treatment group |
| --- | --- |
| nC | Number of observations in the control group. |
| XT | Number of events in the treatment group |
| XC | Number of observations in the control group. |

### Value

A list with 14 components,

| K | Number of studies, |
| --- | --- |
| Q | the standard Q statistic (inverse variance method), |
| p_Q | the p-value from the chi-square approximation with K-1 df |
| E_th(Q) | the estimated value of the mean of Q from theoretical expansion |
| E(Q), var(Q) | The estimated values of the mean and the variance of Q from the improved approximation |
| alpha, beta | shape and scale parameters for the gamma approximation |
| p_gam | the p-value from the gamma approximation |
| miss | Indicator of error (1) for the gamma approximation |
| BD_stat | the Breslow-Day test statistic |
| P_BD | p-value from the Breslow-Day test |
| BDT_stat | the Tarone-corrected Breslow-Day test statistic |
| P_BDT | p-value from the Tarone-corrected Breslow-Day test |
|  |  |

### Details

Log-OR values and percentage weights are calculated for each study. The weights are based on the inverse variances.

The program calculates the standard Cochran’s Q statistic and its p-value based on the chi-square approximation; the estimated value of E_th(Q) from a theoretical expansion; estimates of E(Q) and var(Q) from the improved approximation; the shape (alpha) and scale (beta) parameters for the gamma approximation with these moments, and the corresponding p-value for the Q test of heterogeneity of ORs. The output also includes the Breslow-Day (BD) statistic and its p-value, and the Tarone correction to the BD test, denoted by BDT.

### Required functions

Please, source the file Q_OR_dat.txt which contains the function new_QOR. This function calls the file LOR_moments_final.txt which contains the required functions:

Qfor_LOR

paras_QLOR

paras_QLOR_std

Q_LOR

momLogit_exactth

cmomLOR_th

EQ

breslowdaytest

### Author(s)

Elena Kulinskaya

**References**

Kulinskaya, E. and Dollinger M.B. An accurate test for homogeneity of odds ratios based on Cochran's Q-statistic. *BMC Medical Research Methodology*, 2015

Cochran, W. (1937). Problems arising in the analysis of a series of similar experiments. *JRSS* **4**, 102–118.

Breslow, N.E., Day, N.E.: Statistical methods in cancer research. International Agency for Research on Cancer, 136--146 (1980)

Tarone, R.E.: On heterogeneity tests based on efficient scores. *Biometrika* **72**, 91--95 (1985)

### Examples

# Example with manually read input data

source("LOR_moments_final.txt ")

XT<-c(27,34,53,34,1,28,15,11,12,43,43,77,8,42,11,14,5)

# Number positive in the treatment groups

nT<-c(203,237,468,1031,104,144,292,504,85,577,740,512,114,2199, 208,443, 101)

# Number observations in the treatment groups

XC<-c(28,20,35,8,1,12,5,4,11,17,35,58,5,5,5,13,4)

# Number positive in the control groups

nC<-c(206,234,489,1107,106,106,292,187,78,532,637,549,68,929,216,422,90) # Number observations in the control groups

new_QOR(nT,nC,XT,XC)

# Example with the data read from a *.csv

# source("LOR_moments_final.txt ")

stead<-read.csv("F:Stead.csv", header=T)

new_QOR(stead$nE,stead$nC,stead$xE,stead$xC)
